# Dual-energy CT after radiofrequency ablation of liver, kidney, and lung lesions: a review of features

**DOI:** 10.1007/s13244-015-0408-y

**Published:** 2015-05-05

**Authors:** Frederik Vandenbroucke, Steven Van Hedent, Gert Van Gompel, Nico Buls, Gordon Craggs, Jef Vandemeulebroucke, Pablo R. Ros, Johan de Mey

**Affiliations:** 1 Department of Radiology, UZ Brussel (VUB), Laarbeeklaan 101, Brussels, Belgium; 2 Department of Electronics and Informatics, Vrije Universiteit Brussel, Brussels, Belgium; 3 Department of Medical IT, iMinds, Ghent, Belgium; 4 Department of Radiology, University Hospitals Case Medical Center/Case Western Reserve University, Cleveland, OH USA

**Keywords:** Dual-energy CT, Radiofrequency ablation, Liver, Lung, Kidney

## Abstract

Early detection of residual tumour and local tumour progression (LTP) after radiofrequency (RF) ablation is crucial in the decision whether or not to re-ablate. In general, standard contrast-enhanced computed tomography (CT) is used to evaluate the technique effectiveness; however, it is difficult to differentiate post-treatment changes from residual tumour. Dual-energy CT (DECT) is a relatively new technique that enables more specific tissue characterisation of iodine-enhanced structures because of the isolation of iodine in the imaging data. Necrotic post-ablation zones can be depicted as avascular regions by DECT on greyscale- and colour-coded iodine images. Synthesised monochromatic images from dual-energy CT with spectral analysis can be used to select the optimal keV to achieve the highest contrast-to-noise ratio between tissues. This facilitates outlining the interface between the ablation zone and surrounding tissue. Post-processing of DECT data can lead to an improved characterisation and delineation of benign post-ablation changes from LTP. Radiologists need to be familiar with typical post-ablation image interpretations when using DECT techniques. Here, we review the spectrum of changes after RF ablation of liver, kidney, and lung lesions using single-source DECT imaging, with the emphasis on the additional information obtained and pitfalls encountered with this relatively new technique.

*Teaching Points*

*•Technical success of RF ablation means complete destruction of the tumour.*

*•Assessment of residual tumour on contrast-enhanced CT is hindered by post-ablative changes.*

*•DECT improves material differentiation and may improve focal lesion characterisation.*

*•Iodine maps delineate the treated area from the surrounding parenchyma well.*

## RF Ablation

Minimally invasive therapies are increasingly used in patients with malignant tumours, who are not suitable candidates for surgical resection. The aim of local ablative therapy is to induce cell death. Radio-frequency (RF) ablation has attracted much attention in the last decade because of its technological improvements and has become a well-established technique to treat primary and secondary hepatic malignancies, as well as kidney and lung tumours.

Technical success following RF ablation is due to the complete destruction of the tumour. In case of incomplete necrosis of the tumoral volume, re-ablation of residual tumour can be pursued in order to improve technique effectiveness. For this reason, it is crucial to evaluate the results of the procedure early after ablation. The ideal imaging technique should show the degree of necrosis of the malignant mass and detect residual tumour.

Imaging immediately after ablation also allows for the detection of post-procedural complications and provides a baseline for future follow-up comparisons. Unfortunately, the assessment of residual tumour, usually by means of contrast-enhanced computed tomography (CT), is hindered by post-ablative changes, particularly at the periphery of a treated lesion where blood flow is greatest [1, 2].

On pathology this represents a haemorrhagic rim corresponding to an early inflammatory reaction to the necrotic tissue [3, 4]. Today, contrast-enhanced CT is the standard approach to image early post-ablative changes in the liver [5, 6], kidney [7], and lung [8]. However, some investigators have reported that although complete necrosis may appear clearly on contrast-enhanced CT, these findings do not always correlate with histopathological conclusions, which suggests that there is limited diagnostic accuracy in the detection of residual tumours [9–12].

## Dual-energy CT

A well-known drawback of standard CT is that a number of different materials or tissues may show similar attenuation behaviour at single radiation energy levels and, with that, similar corresponding Hounsfield numbers. This poor attenuation difference can be partially resolved by using dual-energy CT (DECT) technology with material decomposition [13]. DECT is able to achieve tissue attenuation sampling at two different energy spectra, which results in more specific information beyond the typical Hounsfield units.

For already several years, three main technical approaches have been developed by various vendors for the acquisition of dual-energy data: a single-source rapid kilovolt peak-switching technique, a dual-source technique with an angular offset, and finally use of a dual-layer detector that discriminates between high- and low-energy photons [13]. The first two techniques obtain dual-energy data simultaneously at two different energy levels at typically 80 and 140 kVp, although dual-source systems can also operate at 100 and 140 kVp (with an additional tin filter) in case of larger patient sizes. The third technique uses the detected high- and low-energy signal components in one CT acquisition. More recently, a non-simultaneous, single-source technique has been reported that applies a sequential data acquisition and a coregistration motion correction algorithm [14]. It has been shown that DECT improves material differentiation and works especially well in materials with large atomic numbers, such as iodine and calcium, because their strong photoelectric effect causes high attenuation at lower photon energies [15]. The material decomposition technique assumes that for any tissue an equivalent mixture of water and iodine exists with similar spectral attenuation properties. Consequently, after a material decomposition calculation process, two base material maps can be displayed, representing the concentrations of water and iodine in each voxel. In contrast studies, these concentrations correspond to real water/iodine concentrations in blood. All other materials (e.g. bone, fat) are described as a mixture of both of these base materials and will appear as hyper- or hypodensity in the base pair maps. Iodine concentrations can be accurately quantified. A recent study showed a 0.55 mg/cc mean error when comparing calculated and true iodine concentrations in renal masses [16]. These calculated iodine concentrations (mg/cc) can be displayed for any region of interest (ROI) as either a greyscale- or colour-coded iodine image. Such iodine maps obtained from DECT images are not a surrogate for dynamic perfusion CT, as they merely provide a visualisation of the iodine distribution in the tissues at one point in time.

In addition to calculating base material maps, synthesised or synthesised monochromatic images from dual-energy CT, representing CT values (HU) over a range of 40 to 140 keV, can also be obtained. Compared to high-keV, low-keV images (closer to the k-edge of iodine) typically provide improved contrast between different structures, e.g. between a tumour and surrounding normal parenchyma after injection of iodinated contrast material. On the other hand, noise is more prominent in these lower keV images [17]. Synthesised monochromatic images at 77 keV correspond best to the effective energy of a single-energy 120-kV scan [18], such that the image contrast simulates a traditional single-energy CT scan. By assessing the DECT images, an optimised keV window based on the contrast-to-noise ratio between tissues can be calculated. In RF ablation, an improved contrast is typically obtained between the ablation zone and surrounding normal liver parenchyma. A scatterplot analysis, which estimates the material concentration in each voxel, can also help to differentiate different structures in the ROIs. Research is currently ongoing with regard to adequate quantification of the acquired DECT data. Several potential candidate parameters are being screened for usefulness within the RFA context, such as CT numbers (i.e. Hounsfield unit curves) [19, 20], contrast-to-noise ratios [20, 21] or iodine concentrations [22]. Compared to standard CT, DECT may have the potential to improve focal lesion characterisation, and it is a promising tool to supply quantitative data in addition to traditional morphological information. The associated radiation dose with DECT will depend on the applied technology. Although specific clinical studies comparing the dose efficiency of different technologies are still lacking, current data suggest that DECT imaging with dual-source systems does not necessarily cause additional radiation exposure for the patient compared to standard CT [17, 23, 24]. Radiation dose data on the rapid kilovolt peak–switching technique to date are still inconclusive and reports from other approaches are scarce or nonexistent [25]. However, the increased informational content and post-processing flexibility of DECT data create additional opportunities for dose saving, such as the creation of virtual unenhanced images from a contrast-enhanced scan [25].

The purpose of this pictorial essay is to display post-ablative changes on contrast-enhanced CT with a single-source rapid kilovolt peak-switching technique in order to identify the additional information provided by DECT imaging and to describe the pitfalls of this relatively new technique in the evaluation of RF ablation’s technical success.

## DECT imaging protocol

Fast kilovoltage switching CT was performed on a 64-slice CT (Discovery CT750 HD, GE Healthcare, Milwaukee, WI, USA) after IV administration of 120 cc contrast at 2.5 cc/s. Scan data were acquired at 40-mm collimation using predefined GSI protocols at CTDI_vol_ values between 15.02 and 25.53 mGy. Fixed tube currents (between 375 and 600 mA) were used as our DECT scanning mode is not compatible with the automated tube current modulation system of the scanner. Images were processed on a workstation (AW4.4; GE Healthcare) using the Gemstone Spectral imaging application. Three types of images were reconstructed for analysis: synthesised monochromatic images from 40 to 140 keV, greyscale- and colour-coded iodine images (with ‘rainbow’ colour map).

### Liver ablation

**Fig. 1:**
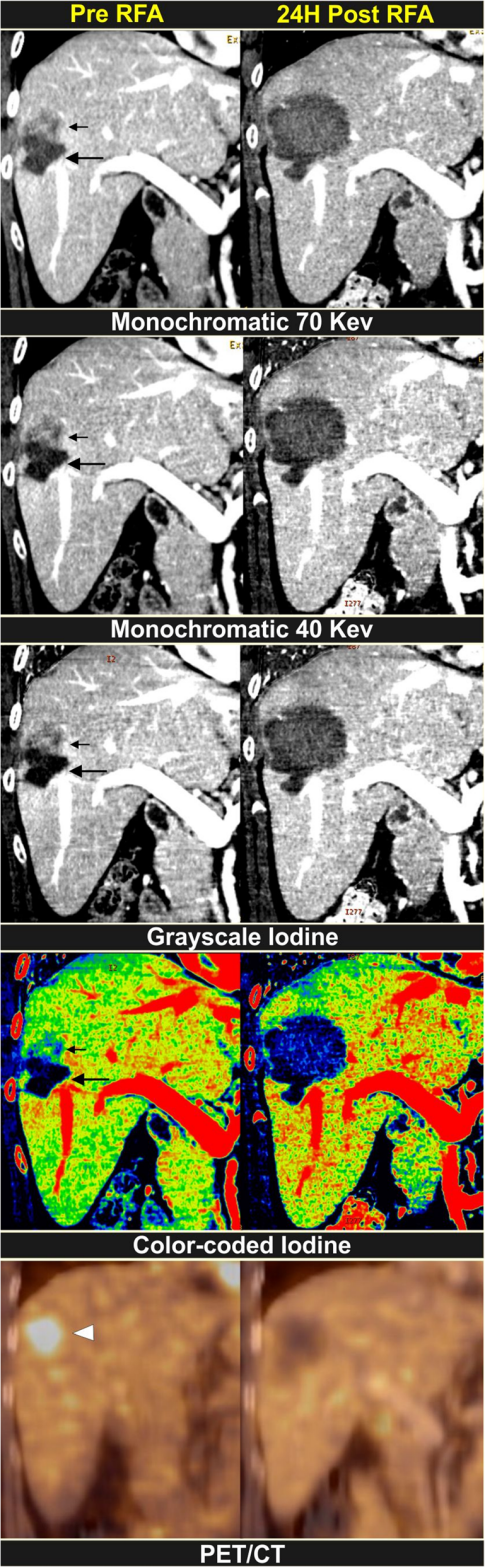
Hypovascular liver metastasis. A 53-year-old female undergoes surgery for rectal carcinoma and then receives RF ablation in a focal liver lesion. Eight months later, a local tumour progression (first column) at the cranial side of the ablation zone (*large arrow*) can be seen as an intermediate density (*small arrow*). In this straightforward case, the tumoral tissue is equally well depicted on the 40- and 70-keV images as on the greyscale iodine images. Noise is more prominent on the 40-keV image however. Iodine maps are encoded using a rainbow template with a colour coding from high to low iodine concentration, being from red (through yellow) to blue respectively. The intermediate colour represents a tumour (*small arrow*). On PET/CT a vivid FDG uptake is seen (*arrowhead*). Twenty-four hours after a second RF ablation (second column), a sharp delineated ablation zone is found. On all reconstructions, the avascular nature of the ablation zone is well demonstrated. The contrast with the surrounding liver parenchyma appears highest on the colour-coded iodine map. No FDG uptake is seen on the PET/CT image. The PET/CT 3 months later (not shown) does not show signs of tumoral activity

**Fig. 2:**
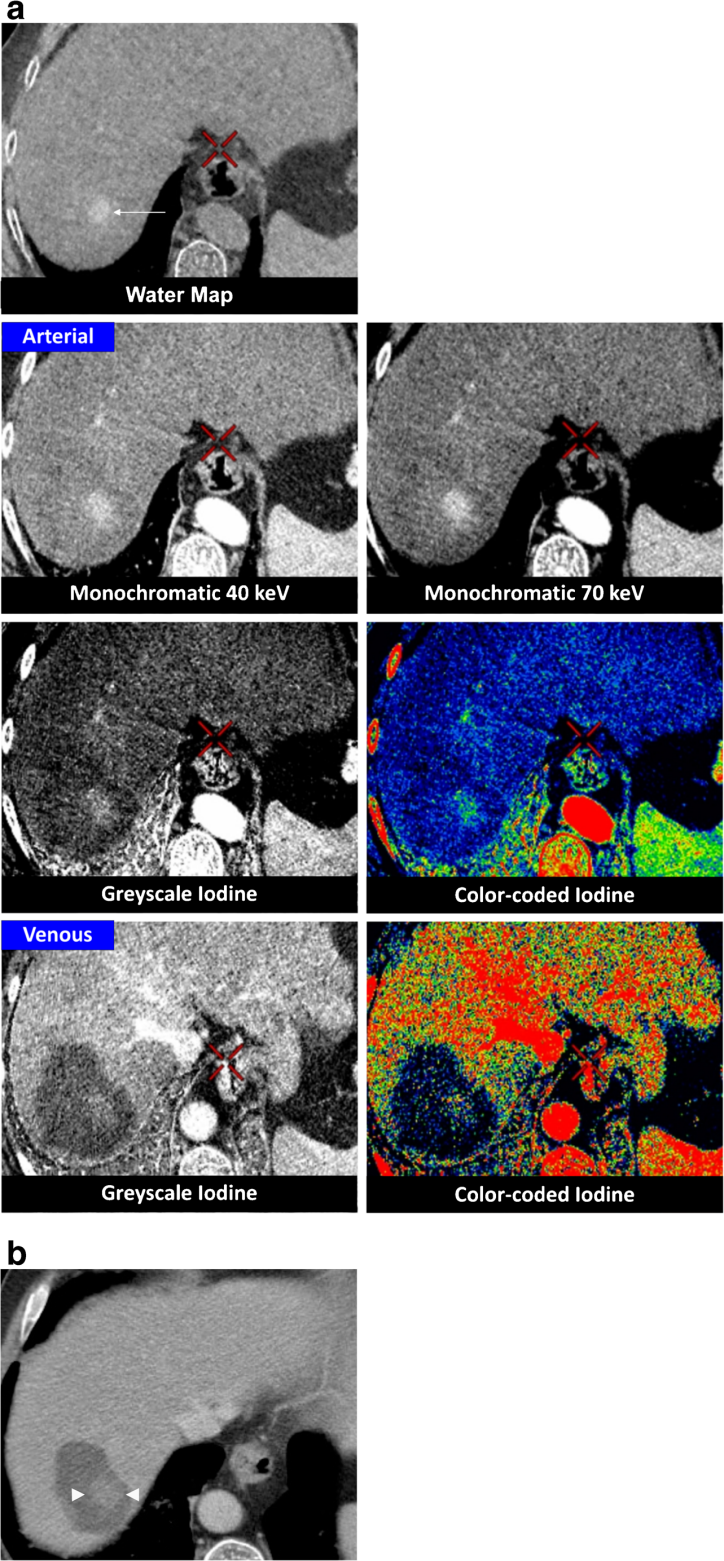
Hypervascular metastasis. A 63-year-old male undergoes follow-up CECT in the arterial phase 4 years after ablation of a renal cell carcinoma. This reveals a 26-mm hypervascular lesion in segment VII of the liver, which is histopathologically proven to be a metastasis of the RCC. RF ablation is performed. **a** Twenty-four hours after the ablation, a DECT is performed. The water map image shows a centrally located hyperdensity in the ablation zone (*arrow*), corresponding to intense charring and desiccation of the treated lesion. Note that this hyperdensity is less visible on the iodine maps. The arterial phase image does not demonstrate any focal hypervascularity at the border of the ablation zone. No rim enhancement can be seen. The delineation of the total ablation zone is better depicted on the portal-venous phase greyscale- and colour-coded iodine images, because the amount of iodine in the liver parenchyma is higher. **b** On the 1-year follow-up CECT, a smooth delineation of the ablation zone is noticed, which serves as proof of the technical success of the ablation. The central hyperdensity however is still noticeable (*arrowheads*)

**Fig. 3:**
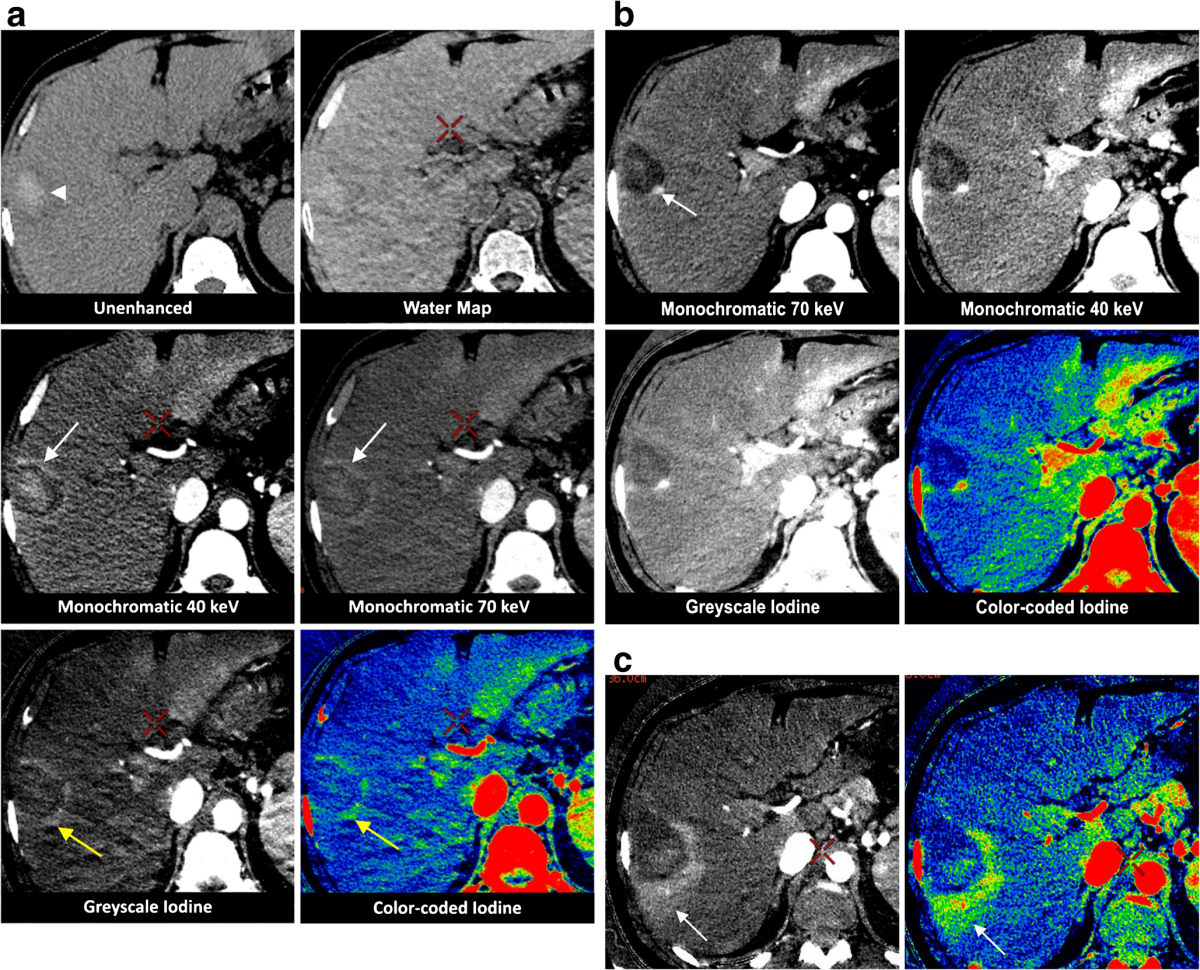
Hepatocellular carcinoma. A 43-year-old male with cirrhosis undergoes gadolinium enhanced MRI, revealing a hepatocellular carcinoma in segment VII, typical for a hepatocellular carcinoma, which is confirmed through biopsy. **a** Twenty-four hours after RF ablation, the patient undergoes a standard unenhanced CT scan and a contrast-enhanced DECT. The unenhanced CT shows a central area of high attenuation (*arrowhead*) within the ablation zone, which has a lower visibility on the water map reconstruction. The central hyperdensity is well noticeable on the synthesised monochromatic 40-keV but less so on the 70-keV images, and it is hardly visible on the iodine images. On the 40- and 70-keV synthesised monochromatic images, the ablation zone can be depicted as a lesion with a hypervascular peripheral rim (*white arrows*). On the greyscale- and colour-coded iodine images, we can better appreciate the focal hypervascular thickening at the posterior border (*yellow arrows*) than with the synthesised monochromatic images. **b** Due to the suspicious nature of the focal thickening seen in **a**, a shorter follow-up period is adhered to and a DECT is performed 5 weeks post-RF ablation. A focal hypervascular nodule is well depicted on the 70-keV (*white arrow*) images, although more clearly observable on the 40-keV ones; however again it is more evident on the greyscale- and colour-coded iodine images. Biopsy of this region confirms residual HCC. **c** Twenty-four hours after the second ablation, the greyscale- and colour-coded iodine images show significant iodine rim uptake around the ablation zone in the arterial phase (*arrows*). Therefore, a focal hypervascular remnant cannot be appreciated. This rim disappears on the venous phase (not shown). MRI after 10 weeks reveals no signs of reactivation

Necrosis caused by thermal damage is characterised by an absence of blood perfusion, resulting in a non-enhancing area on contrast-enhanced CT (Table 1). Due to this lack of internal vascularity, iodine maps are well suited for delineating the treated area from the liver parenchyma (Figs. 1 and 2a). Dual-energy CT has the possibility to extract iodine from the enhanced images to create water map images and thus potentially skip true unenhanced images, as shown in Fig. 3a. This undoubtedly offers a considerable advantage in limiting radiation dose exposure [23]. However, a drawback is that current DECT techniques do not produce water map images with the same contrast-to-noise ratio as true unenhanced images [26].

**Table 1:** Early and long-term findings after RF ablation of liver lesions in successful and unsuccessful results, with the added value of the DECT technique

		Early findings	Long-term findings
Successful	General findings	Non-enhancing area on contrast-enhanced CT Regular hypervascular peripheral rim Central hyperattenuation is indecisive for success	Regular borders of the ablation zone Reduction in size
	Added value of DECT	Iodine void area Improved contrast on the 40-keV images Excellent conspicuity of the ablation zone on iodine-coded images	Sharp depiction of the ablation zone’s edge on iodine images
Unsuccessful	General findings	Irregular rim Focal nodular thickening at the border	Focal hypervascular nodule (HCC) Nodular attenuation differences around the ablation zone (hypovascular metastasis)
	Added value of DECT	Higher residual tumour-to-liver contrast on iodine images Increased attenuation of iodine on the low-keV images for detection of subtle residual tumour	Better contrast between LTP and liver parenchyma on iodine images

Frequently, a hyperattenuation centrally within the ablation zone can be seen on unenhanced images [27]. It is thought that this area of high density correlates to a region of greater cellular disruption in the centre of the ablation zone [3], reportedly caused by the intense charring of the severely desiccated coagulated lesion [28]. It has usually disappeared by the time the next follow-up CT examination takes place, but can persist for a longer time [29] (Fig. 2b). This hyperattenuation is observable on both the water map and true unenhanced images. Despite the absence of iodine, this can also be seen on the iodine-coded images, since the hyperattentuated area is represented as a combination of both water and iodine after material decomposition (Fig. 2a). Meticulous comparison of water and iodine-coded images allows for differentiation of this central hyperattenuation after RF ablation and structures enhancing after IV contrast administration. The reader should however be aware of the lower image quality of water images with respect to true unenhanced images (Fig. 3a), as also reported on dual-source DECT [20]. Similar to the study of Lee et al. [20], we observed sharp depiction of the edge of the ablation zone of the iodine images. Contrary to their findings obtained using a dual-source DECT system, our experience with single-source DECT images did not prove an internal homogeneity of the ablation zone on the iodine images.

Immediately after ablation, it is common to find a hyper-attenuating halo surrounding the ablation zone on CECT, correlated to an increase in arterial perfusion due to capillary leakage from thermal damage [30]. The circumferential enhancement is predominantly visible on the arterial phase and can be very prominent in some cases (Fig. 3c), obscuring the interpretation of residual tumour. An irregular or nodular rim can indicate the presence of residual tumour, although benign variations do occur [30–32]. When focal thickening at the border of the ablation zone is unclear, a shorter follow-up period is recommended [32]. Previous studies have shown that a hypervascular rim remains visible in 89 % of cases after 1 month, in 56 % of cases between 1 and 3 months, and in 22 % of cases between 3 and 6 months [33]. Ideally, the volume of thermal necrosis exceeds the limits of the metastasis by 1 cm in all dimensions [34]. Residual tumour will persist as a focal nodular enhancement in the periphery of the ablation zone (Fig. 3b). For dual-energy CT, the increased attenuation of iodine on the low-keV (e.g. around 40 keV) images is better suited for detecting any subtle density differences around the ablation zone (Fig. 3b). Adding colour-coding enhances the visibility of the contrast in the image.

The water-iodine material decomposition can highlight areas containing iodinated contrast. Iodine maps are superior to synthesised monochromatic images for the qualification of contrast uptake. Consequently, a hypervascular nodule with a higher inflow of iodine is better depicted on the iodine maps when compared to synthesised monochromatic images (Fig. 2b).

### Kidney ablation

**Fig. 4:**
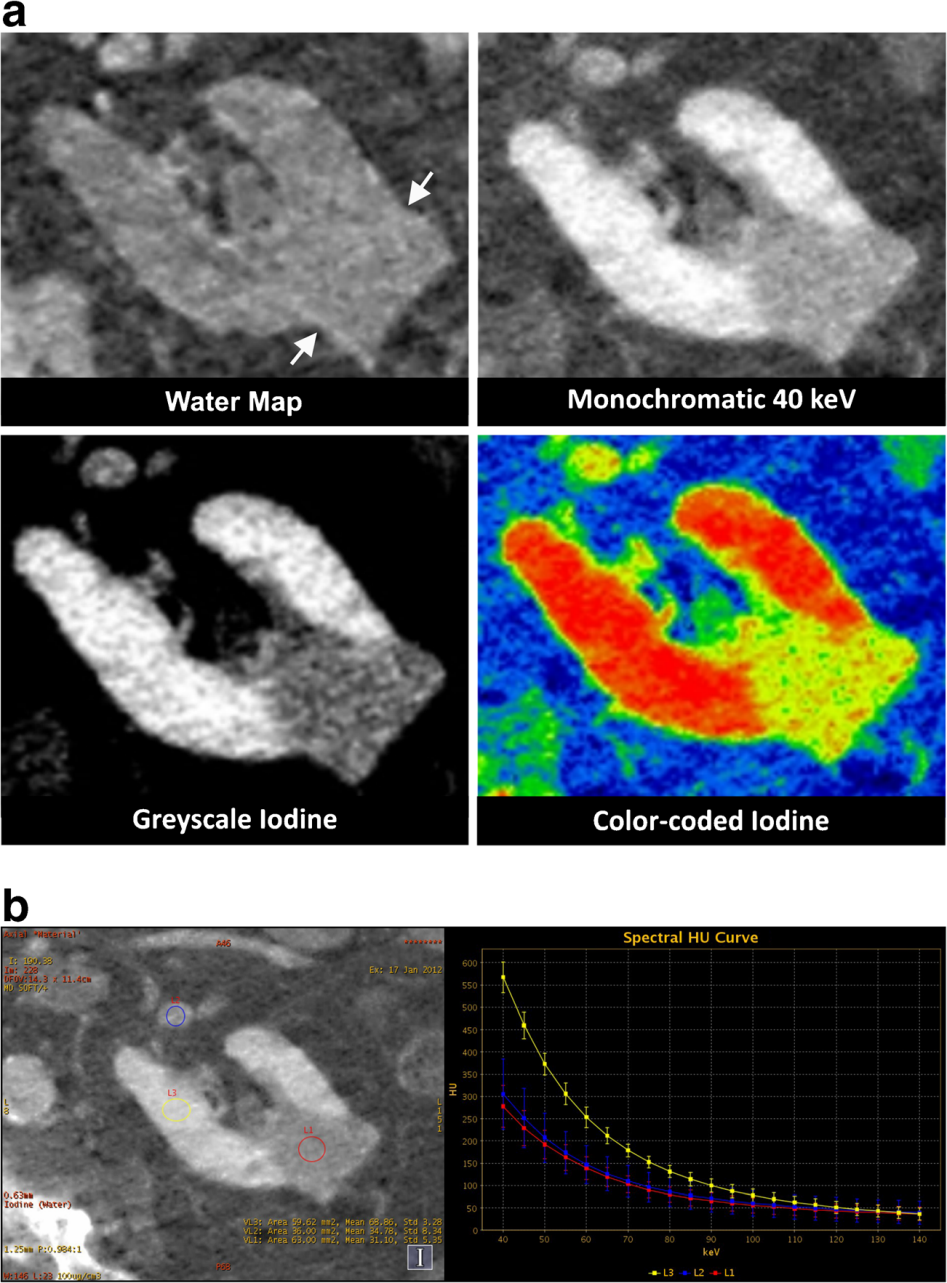

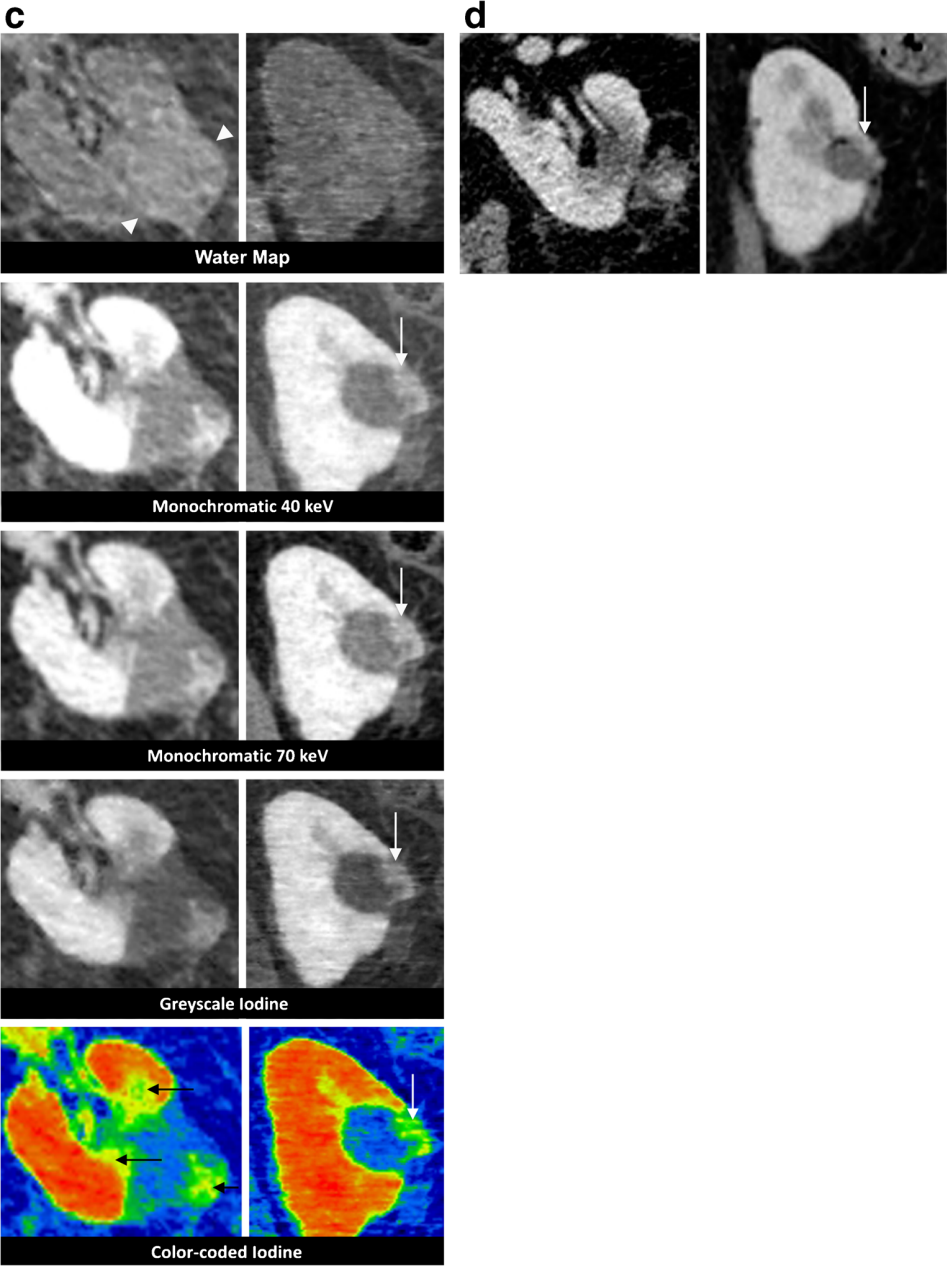
Renal cell carcinoma. An ultrasound reveals a renal mass in a 72-year-old male. **a** Venous phase DECT confirms a lesion in the left kidney. On the water map images, the lesion is exophytic and isodense compared to the kidney parenchyma (*arrows*). The synthesised monochromatic 40-keV greyscale-coded and colour-coded iodine images show a hypovascular lesion compared to the kidney parenchyma. A biopsy demonstrates a renal clear cell carcinoma. **b** On the iodine-coded image (left), three ROIs are selected: L1 (red) in the RCC; L2 (blue) in the renal vein; L3 (yellow) in the unaffected kidney parenchyma. The estimated amounts of iodine in the ROIs are presented in the right lower corner (mean value and standard deviation in 100 μg/cm^3^). On the spectral HU curve (right), the renal vein (L2) and RCC (L1) represent overlapping curves, suggesting similar iodine content. The normal kidney parenchyma (L3) has a markedly higher curve in the lower keV range, confirming a higher iodine uptake. **c** Axial (left column) and coronal (right column) reconstructions from a contrast-enhanced venous phase DECT, 24 h post-RF ablation. The water map images show very poor hyperattenuation in the ablated zone (*arrowheads*). The contrast difference between the ablation zone and the kidney parenchyma is well demonstrated on the synthesised monochromatic 40- and 70-keV images. The colour-coded iodine images show well-depicted areas of intermediate density on the axial reconstructions (*black arrows*) surrounding the ablation zone. The axial images of the synthesised monochromatic reconstructions less clearly visualise these regions. The multiplicity suggests a benign post-ablative finding, probably corresponding to transient thermal damage to the bordering kidney parenchyma. No definitive conclusion can be made concerning residual tumour solely relying on these images. However, compared to the pre-ablation imaging, we can assume that the tissue in question is a normal kidney parenchyma. At the outer margin of the ablation zone, a region of intermediate density is depicted. The coronal reconstructions are essential to prove the continuity with regard to the kidney parenchyma (*white arrows*). **d** On the follow-up CECT 15 months later, the focally enhanced tissue at the lateral side shows no signs of growth (*arrow*), thus confirming the absence of residual tumour

Ablation zones in the kidneys lack iodine content and are often wedge shaped because of peripheral infarctions [35] (Table 2). Determining a tumour’s iodine concentration before RF ablation can provide a baseline for future follow-up assessments (Fig. 4a, b). Iodine concentrations that are similar to the original tumour post ablation can be a sign of local tumour progression. The iodine content can be assessed qualitatively as well as quantitatively in the investigated ROI (Fig. 4b).

**Table 2:** Early and long-term findings after RF ablation of kidney lesions in successful and unsuccessful results, with the added value of the DECT technique

		Early findings	Long-term findings
Successful	General findings	Non-enhancing area on the contrast-enhanced CT Often wedge shaped because of peripheral infarctions Streaky soft tissue attenuations in the perirenal fat	Regular borders of the ablation zone Slow reduction in size Fat between the ablation zone and normal kidney parenchyma
	Added value of DECT	Improved contrast on the 40-keV images Clear differentiation between avascular and viable tissues on iodine images	Sharp depiction of the ablation zone’s edge on the iodine images
Unsuccessful	General findings	Persistent enhancement at the border of the ablation zone	Nodular attenuation difference around the ablation zone
	Added value of DECT	Iodine concentrations that are similar to the original tumour, with quantitative assessment Higher lesion-to-liver contrast on the iodine images	Intermediate iodine concentration between the ablation zone and normal kidney parenchyma at the level of the tumour

When evaluating the contrast uptake in a lesion, it is imperative to compare the lesion’s density on the water map images to that on the synthesised monochromatic images in order to be able to appraise possible ‘enhancement’. The iodine content can be assessed qualitatively based on the iodine-coded images as well as quantitatively when expressed in iodine concentrations (mg/ml) (Fig. 4b) in the investigated ROI. Enhancement can also be demonstrated by comparing the iodine concentration in the lesion to a surrounding vascular structure after an IV contrast injection. When evaluating colour-coded iodine maps, it is important to compare iodine concentrations in the ROI to those in adjacent structures, because windowing (as a diagnostic tool) causes variability in the colour spectrum.

**Fig. 5:**
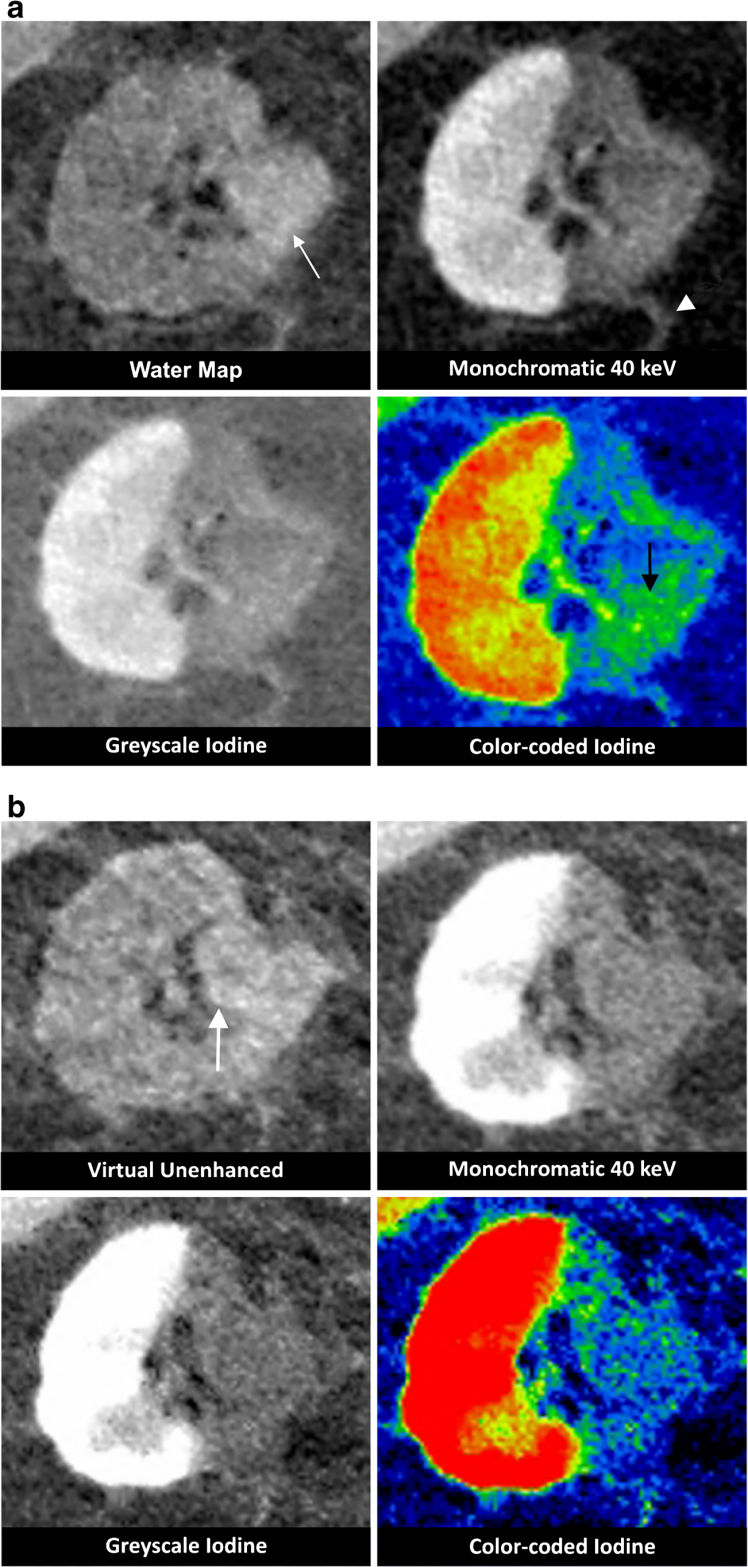
Renal cell carcinoma. A lesion in the right kidney of a 73-year-old male is confirmed as being a hypernephroma through biopsy. The patient is treated with RF ablation, while cooling the pyelum with a double j-stent. **a** The DECT obtained 24 h post ablation shows a hyperdense region in the ablation zone on the water map images (*white arrow*). On the synthesised monochromatic 40-keV images, a sharp delineation of the ablation border is assessed, without the presence of any internal hyperdensity in the ablation zone. A post-procedural, streaky, soft-tissue attenuation can be seen in the surrounding perirenal fat (*arrowheads*). The avascular zone is well depicted on the greyscale- and colour-coded iodine images. In this window level, some yellowish coloured pixels are seen in the centre of the ablation zone on the colour-coded iodine images (*black arrow*), lying in the extension of a branch of the renal artery, explainable as being spilled iodine due to arterial perfusion. **b** DECT reconstructions at a 3-month follow-up stage show an overall stability in the size of the ablation zone. The ablated zone is still slightly hyperdense on the water map image (*arrow*). There is no uptake of iodinated contrast on the greyscale- and colour-coded iodine images, which proves that this area is completely avascular and confirms that the colour pixels seen in **a** were not a sign of tumour activity

As with liver lesions, the extent of necrosis in the ablation zone can be assessed more accurately on 40-keV rather than on 70-keV synthesised monochromatic images. Also, the iodine maps show clear differentiation between avascular and viable tissues. Adjacent to the avascular zone, regions of intermediate vascularity can be seen that appear most pronounced on colour-coded iodine images (Fig. 4c). This may correspond to transient thermal damage to the adjacent kidney parenchyma, causing a temporary blood flow decrease in this area. Multi-planar reconstructions are vital in the assessment of questionable regions after the RF ablation procedure (Fig. 4c). Although avascularity is by definition a sign of successful ablation, the ablation zone can show remnants of iodine concentration, possibly due to the extravasation of iodine which itself is caused by vascular damage (Fig. 5a).

**Fig. 6:**
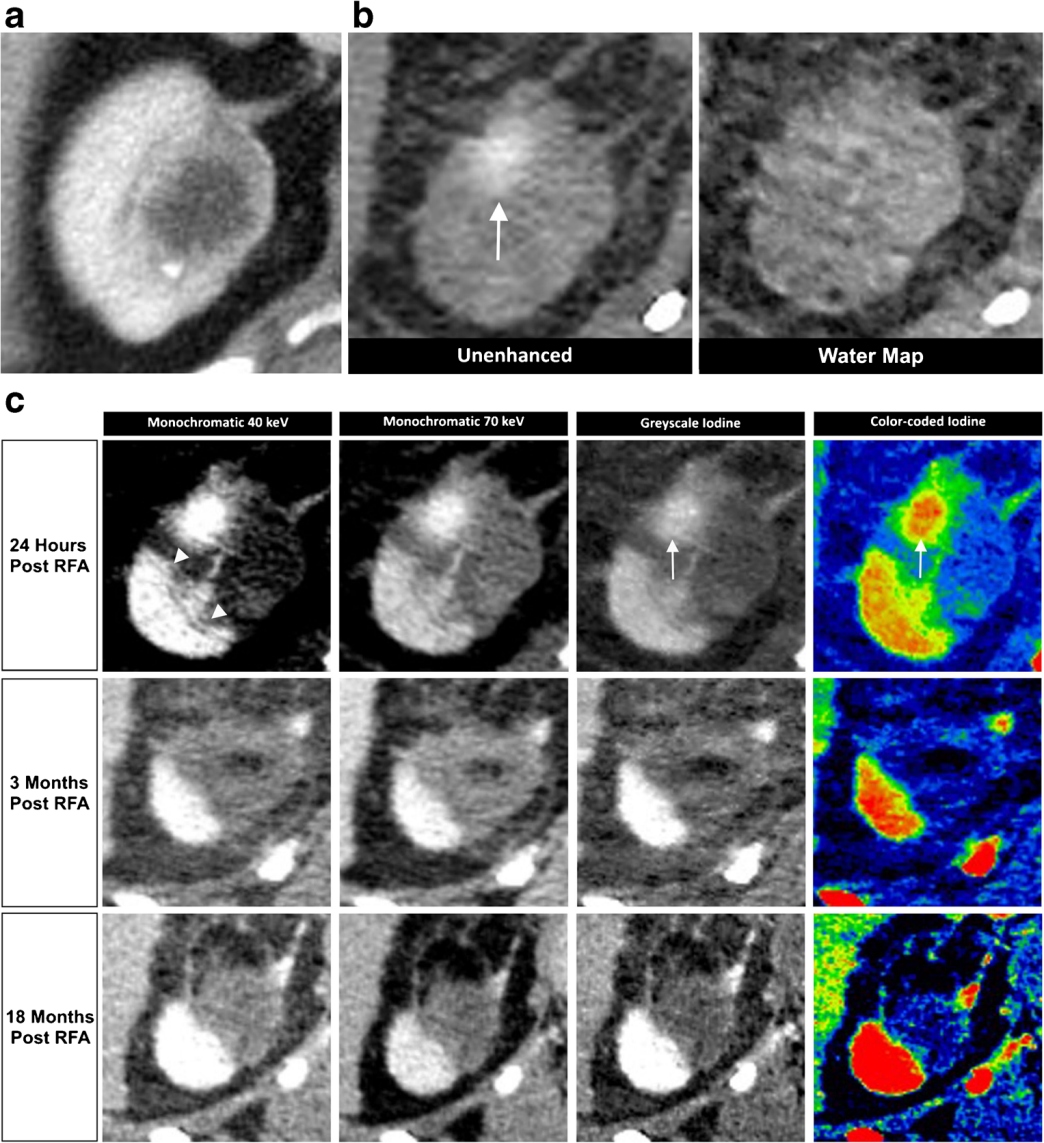
Renal cell carcinoma. A 40-year-old female is diagnosed with a suspicious lesion in the right kidney on ultrasound. After consulting her urologist, the patient opts for RF ablation. **a** CECT shows a thick-walled cystic lesion in the right upper pole, graded as a Bosniak 4 cyst. **b** The true unenhanced polychromatic CT image (left), 24 h post ablation, shows a hyperdense change in the ablation zone (*arrow*). This finding cannot be confirmed on the water map reconstructions. **c** The imaging appearance of the ablation zone 24 h (*first row*), 3 months (*second row*), and 18 months (*third row*) after RF ablation. On the 24-h post-ablation series, the delineation of the ablation zone is clearly observable on all reconstructed images; however, the 40 keV image is of superior quality when compared to the 70-keV image (*arrowheads*). On the iodine-coded images, the internal high attenuation is unexpectedly (no iodine content) still visible within the avascular zone (*arrows*). No suspicious nodular enhancement is observed at the border of the ablation zone. DECT from 3- and 18-month follow-up depicts an involution of the avascular zone. There is a loss of the internal high-attenuation changes. There is no evidence of local tumour progression

The majority of ablation zones are hypodense in nature; however, it is not uncommon to have a variable degree of haemorrhagic necrosis in the necrotic area. This degree of attenuation is not predictive of technique effectiveness (Fig. 6c). Since the quality of the water maps is less compared to the true unenhanced images, spontaneous hyperdensity may be missed by this reconstruction technique (Fig. 6b).

In the contrast to rim enhancement that is commonly found in liver ablations, this phenomenon has only been observed occasionally in ablated renal parenchyma [25]. If present, it resolves quickly and is only marginally visible after 3 months [36]. Post-ablative changes in the perirenal fat are predominantly seen as streaky soft tissue attenuations (Fig. 5a), which resolve to a dominant band or halo over time (Fig. 4d). Long-term follow-up findings include the development of fat between the ablation zone and normal kidney parenchyma [37, 38] (Fig. 4d). The majority of kidney ablations show little to no reduction in size within the first 6 to 12 months [39, 40]; however, earlier shrinkage is not uncommon (Fig. 6c).

### Lung ablation

**Fig. 7:**
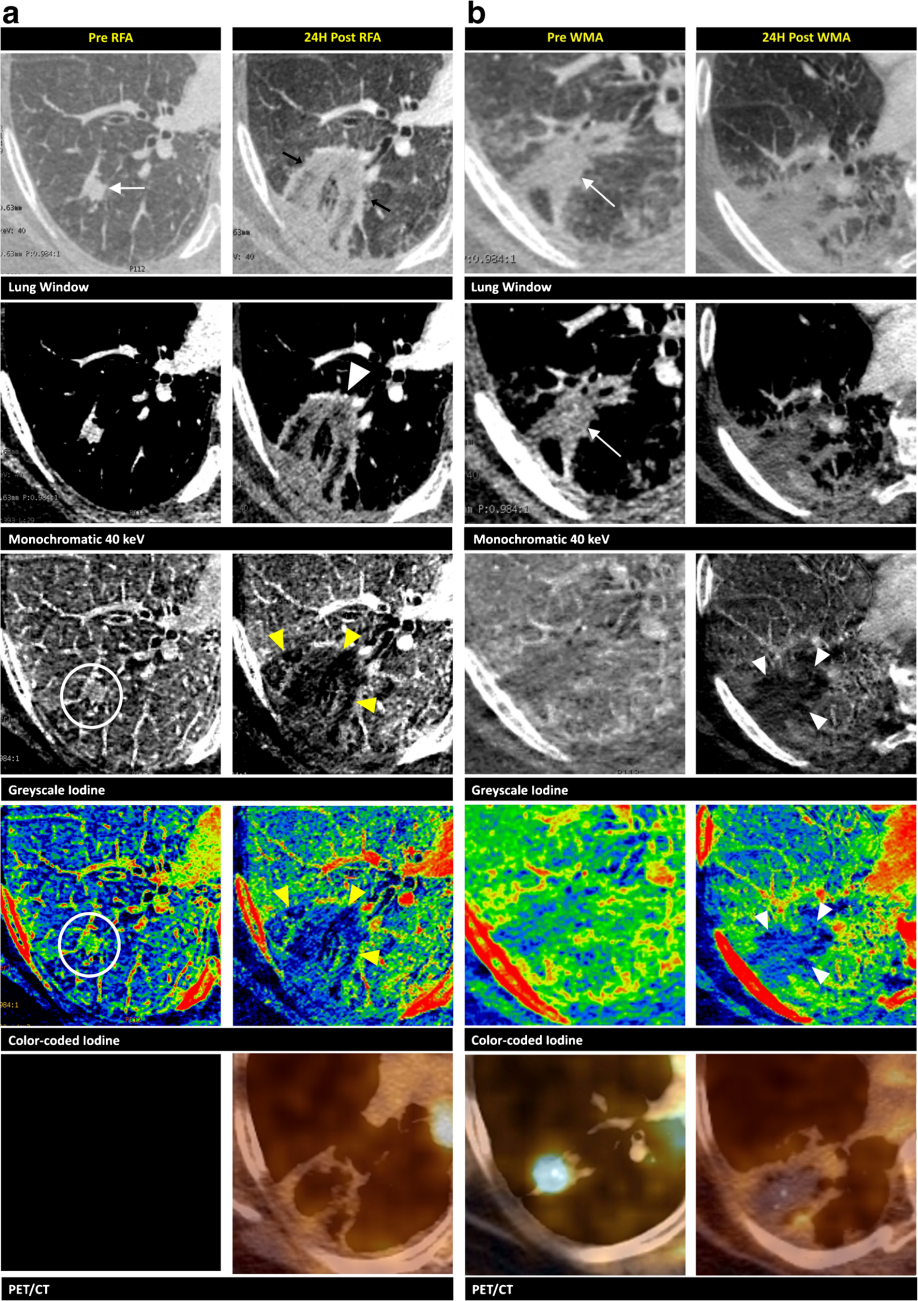
Pulmonary metastases. A 77-year-old female, with a known melanoma, undergoes a PET/CT that shows a hypermetabolic nodule in the right upper lobe (not in our possession). The biopsy is non-conclusive. The therapeutic option is RF ablation. **a** Imaging is performed before (first column) and 24 h after (second column) RF ablation. The 70-keV lung window shows a focal nodule of 11 mm (*white arrow*) in proximity of a blood vessel. This nodule is hyperdense on the synthesised monochromatic 40-keV images and shows iodine content on the greyscale- and colour-coded iodine maps (*circles*). Destructive lung parenchyma is seen after the ablation as an irregular hyperdensity on the lung window (*black arrows*). The synthesised monochromatic 40-keV reconstructions show very heterogeneous densities in this region. The vessel on the anterior side is still intact (*white arrowhead*). On the iodine maps, we clearly observe the absence of iodine uptake (*yellow arrowheads*). PET/CT shows no activity in this region. **b** Imaging is performed before (first column) and 24 h after (second column) microwave ablation (MWA). After 6 months, a well-circumscribed consolidation is seen on the 70-keV lung window and synthesised monochromatic 40-keV image (*arrows*). The greyscale- and colour-coded iodine maps show no focal iodine uptake. This consolidation is hypermetabolic on PET/CT. As a result of the discrepancy between the morphologic and metabolic imaging methods, and the proximity of the blood vessel, which could lead to a heat-sink effect, the patient opts for further focal thermal treatment through microwave ablation. Twenty-four hours after ablation, a large heterogeneously shaped density is recognised on the lung window. The greyscale- and colour-coded iodine maps very clearly show the zone without internal contrast uptake (*arrowheads*) when compared to the 40-keV images. Again, a photopenic area is seen on the PET/CT image

**Fig. 8:**
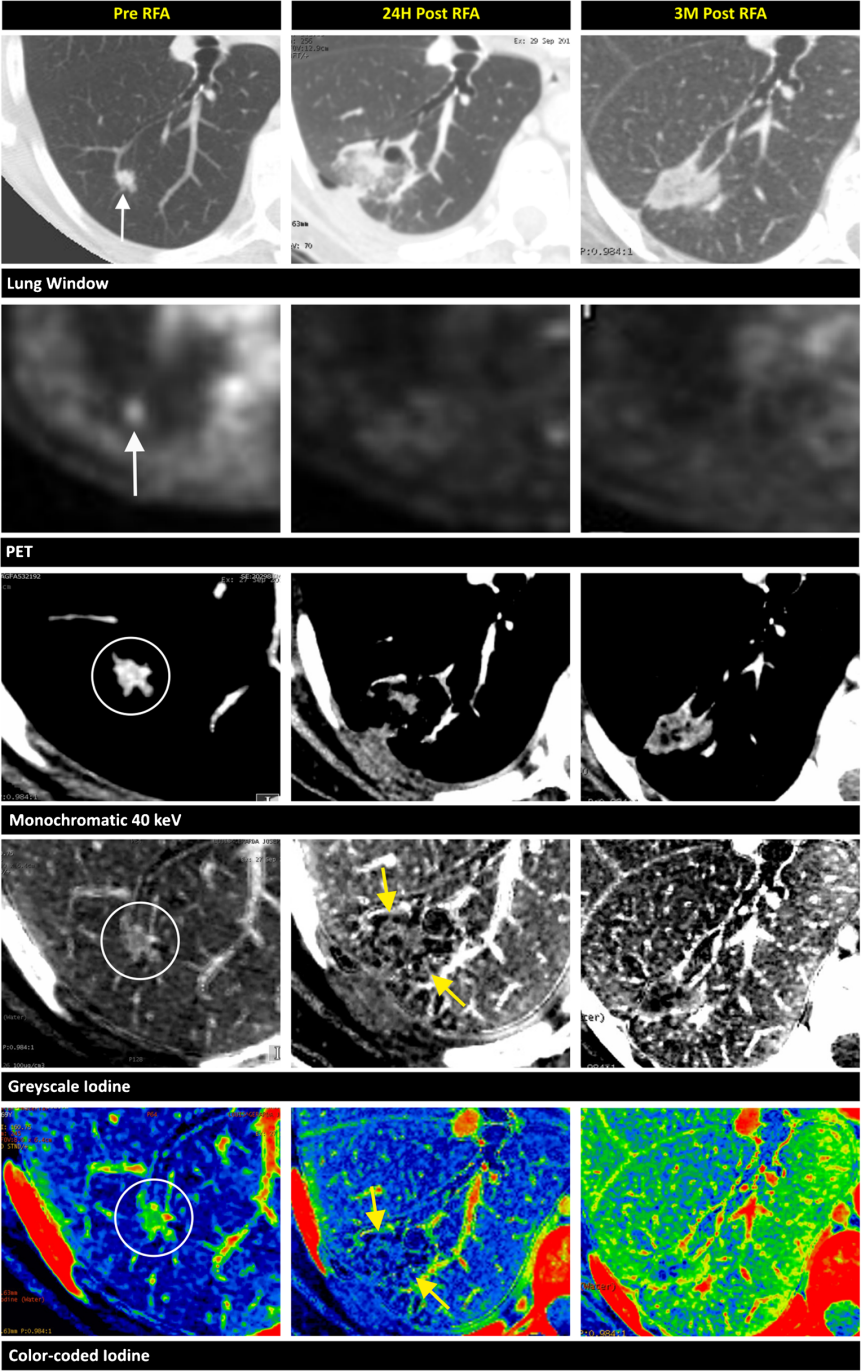
Pulmonary metastases. A 69-year-old female, with a history of breast cancer diagnosed 9 years prior, shows a hypermetabolic nodule in the inferior lobe of the right lung on PET/CT. A biopsy reveals a metastasis of a ductal mammary carcinoma. An RF ablation is performed. Imaging of the lesion 1 day before (first column), **24 h** after (second column), and 3 months after (third column) complete RF ablation is performed. Before ablation, an 8-mm well-circumscribed nodule is depicted on the synthesised monochromatic 70-keV lung window, FDG-avid on the PET scan (*white arrows*). Internal vascularisation is clearly observable on the contrast-enhanced, synthesised monochromatic 40-keV, greyscale- and colour-coded iodine images (*circles*). The changes found 24 h post ablation are a ground-glass appearance (*yellow arrowheads*) around the lesion and the vanishing of the previously seen iodine uptake in the lesion. The post-ablative avascular zone surrounding the necrotic lesion can be delineated on the iodine images as darker areas (*yellow arrows*). On the lung window images after 3 months, the treated area has transformed into a homogeneous, well-delineated area of high attenuation. PET/CT images reveal no activity after the RF ablation. The greyscale- and colour-coded iodine images show a reduction in size of the avascular region

When performing RF ablation of lung lesions, the goal is to achieve an avascular region, void of any iodine enhancement (Table 3). Dual-energy CT in the lungs can depict an abnormal blood flow distribution, improving the detection of acute pulmonary embolism [41]. In addition, we believe that the complete avascular ablative area post ablation is well depicted on the iodine-coded images as an iodine-void area. Therefore, iodine mapping may be an excellent method to evaluate the technique effectiveness and also delineates a more realistic assessment of the necrotic zone after ablation (Fig. 7a). On CECT imaging, the absence of iodine uptake in an ablated lung lesion is hard to identify since the boundary between the ablated and non-ablated tissue cannot be clearly defined (Fig. 8).

**Table 3:** Early and long-term findings after RF ablation of lung lesions in successful and unsuccessful results, with the added value of the DECT technique

		Early findings	Long-term findings
Successful	General findings	Partial or circumferential ground-glass opacification surrounding the treated tumour	Reduction in size
	Added value of DECT	Vanishing of the iodine uptake in the treated tumour, with a clear delineation of the avascular zone on the iodine-coded images	Shrinkage of the avascular zone
Unsuccessful	General findings	Lack of ground-glass opacification around a part of the tumour is not decisive of success	Centrally located enhancements - Enhancement not already present immediately after the RF ablation Growth of the ablation zone Change from ground-glass to solid opacity
	Added value of DECT	Improved contrast of iodine in the lesion on the 40-keV and iodine-coded images ‘Enhancing’ of a part of the tumour on the 40-keV compared to the water map image	Internal iodine uptake in the ablation zone

**Fig. 9:**
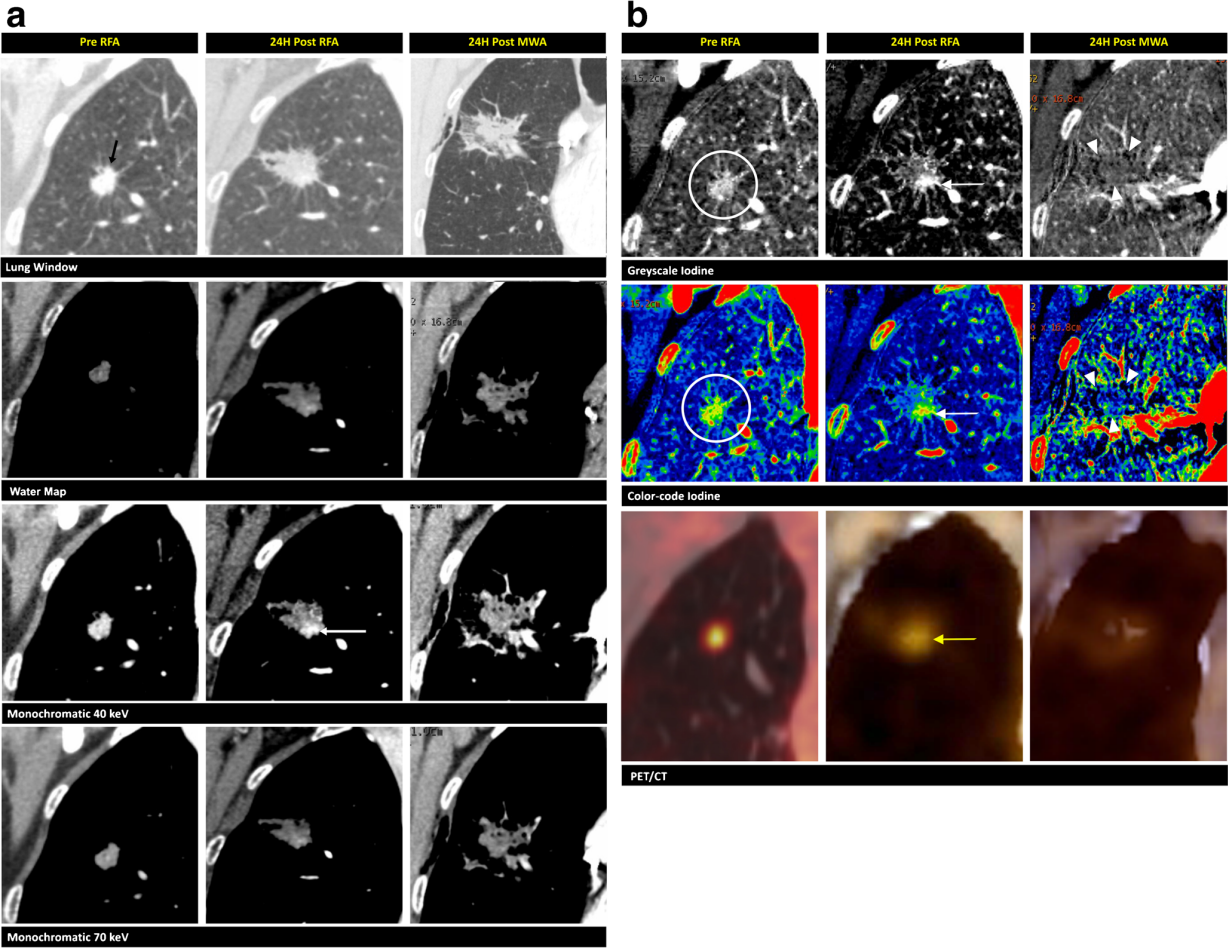
Pulmonary metastases. A focal lung nodule is found in a 65-year-old female diagnosed with ovarian carcinoma. A biopsy reveals a non-small-cell carcinoma. A multidisciplinary team meeting leads to the choice for RF ablation treatment. Coronal reconstructions of the lesion in the right upper lobe before (first column), 24 h after the first RF ablation (second column), and 24 h after microwave ablation (third column) are demonstrated. **a** A focal nodule of 9 mm in the upper lobe is seen on the 70-keV lung window (*black arrow*) prior to the ablation. This nodule is “enhanced” on the synthesised monochromatic images compared to the water map image. This “increase in density” is better appreciated on the 40-keV rather than on the 70-keV images. Twenty-four hours after the first ablation, the spicular nodule appears enlarged on the lung windows. On the 40-keV image, a focal enhancement (*arrow*) is noted at the inferior border of the ablation zone, again more clearly visible compared to the 70-keV image. After re-ablation with microwave technique, we notice more important changes on the lung window than after the first ablation. **b** The iodine content of the hypermetabolic lesion before the RF ablation is most observable on the greyscale- and colour-coded iodine images (*circles*). The nodule is hypermetabolic on PET/CT. Twenty-four hours after the first ablation, the greyscale- and colour-coded iodine maps clearly show the focal area of iodine uptake (*white arrows*) on the inferior border. PET/CT confirms hypermetabolic remnants (*yellow arrow*). After re-ablation, the larger area of thermal damage has lost its internal focal iodine uptake, which is most appreciable on the iodine-coded images (*arrowheads*). Images after the re-ablation lack FDG uptake on PET/CT

The typical finding on lung window CT during and after RF ablation is ground-glass opacification surrounding the treated tumour, which is fully circumferential (Fig. 8) in the majority of cases, but can also be partial (Fig. 9a). It is less common to find ground-glass opacification along the electrode tract. These ground-glass changes typically lead to an overestimation of the necrosis zone by 2–4 mm [42]. The distinction between the inner necrotic zone and the outer haemorrhagic viable zone is not clear on CT imaging; thus, the effective necrotic zone is easily overestimated [8]. As a result, the boundary between the ablated and non-ablated zones cannot be clearly defined using traditional morphologic imaging (Fig. 7b).

It is unclear how the iodine images provide a more realistic view of the necrotic zone after ablation. Kawai et al. [43] evaluated the feasibility of distinguishing ground-glass opacification in adenocarcinomas from that in a haemorrhage or inflammation. They found that there was an increased iodine-related attenuation in adenocarcinomas, but not in a pulmonary haemorrhage or in inflammatory changes. These findings led us to investigate the possible benefits of using iodine maps to distinguish residual tumours (Fig. 9) from the common post-ablative changes found in lung ablation. On follow-up CECT, centrally located enhancements or enhancements that were not present immediately after the RF ablation are indicative of malignancy [44]. Even without enhancement, growth of the ablation zone (after 8 to 10 weeks) or any peripheral nodule is a cause for concern. A change from ground-glass opacity to solid opacity also requires further investigation [43]. Positron emission tomography/CT is considered the optimal follow-up tool after focal ablation in the lung. Residual disease can be detected through tracer uptake, most likely at the periphery of the ablation zone (Fig. 9b). On follow-up, increased or new metabolic activity located centrally (Fig. 7b) or at the outer rim of the ablation zone is a sign of progressive tumour activity [44].

## Conclusion

Post-ablative changes can hamper the evaluation of post-ablation zones. Our pictorial review illustrates the potential improvements that DECT provides in the differentiation of tissues. We believe that DECT can be a valuable asset in the differentiation of residual tumours from benign inflammatory changes, commonly found after the ablation of liver, kidney, and lung tumours. Further investigation is required because of the limited clinical experience with this relatively new technique.
